# Clinical management of indeterminate thyroid nodules needs to be revisited. New evidence for a personalized approach to the problem

**DOI:** 10.1007/s40618-024-02510-3

**Published:** 2024-12-04

**Authors:** Tommaso Piticchio, S. Wolde Sellasie, F. D’Arrigo, F. Galeano, I. Barca, A. Prinzi, R. Le Moli, L. Scappaticcio, S. Amendola, L. Guidobaldi, I. Nardone, S. Zaccaria, F. Pallotti, L. Uccioli, Frasca F.

**Affiliations:** 1 Endocrinology Section, Department of Clinical and Experimental Medicine, Garibaldi Nesima Hospital, University of Catania, Catania, Italy; 2 Department of Medicine and Surgery, University Kore of Enna, Enna, Italy; 3 Division of Endocrinology and Diabetes, Department of Biomedicine and Prevention, CTO Andrea Alesini Hospital, University of Rome Tor Vergata, Rome, Italy; 4 PhD School of Applied Medical-Surgical Sciences, University of Rome Tor Vergata, Rome, 00133 Italy; 5 Unit of Endocrinology and Metabolic Diseases, AOU University of Campania “Luigi Vanvitelli”, Naples, Italy; 6 Department of Advanced Medical and Surgical Sciences, University of Campania “Luigi Vanvitelli”, Naples, Italy; 7 UOC of Pathologic Anatomy and Cytodiagnostic, Sandro Pertini Hospital, Rome, ASL RM2, 00157 RM Italy

**Keywords:** Thyroid nodule, TIR3A, AUS, Indeterminate nodule, Malignancy, Follow-up

## Abstract

**Purpose:**

Thyroid nodules diagnosed by fine needle aspiration cytology (FNAC) as TIR3A or Class III subgroup “other types of atypia” (indeterminate thyroid nodules - ITNs), are the only ones without a unique clinical action indicated for management. This leads to multiple FNAC repetitions (FNAC-reps) and lifelong follow-up, with huge consumption of time and resources. The aims of the study were to inquire the usefulness of repeating FNAC in ITNs and perform an evaluation of a long-term follow-up of a large cohort of ITNs.

**Methods:**

The study was conducted in two Italian high-volume tertiary centres. We selected patients who underwent the first FNAC with subsequent diagnosis of ITN in a centre involved and who then repeated it in the same institute.

**Results:**

We included 506 patients. The FNAC-rep determined the “change in management indications” (CMIs) in 30 cases. The binomial test showed that this proportion was not significant (p 0.36). The factors related to CMIs were age (OR = 0.97; 95%CI = 0.95–0.99; *p* = 0.04), margins (OR = 5.6; 95%CI = 1.7–18.1; *p* = 0.004), and echogenicity (hypoechoic vs. isoechoic: OR = 5.2; 95%CI = 1.87–14.5; *p* = 0.002| hypoechoic vs. iso-anechoic: OR = 5.9; 95%CI = 1.32–26.2; *p* = 0.02). Follow-up of cases without CMIs showed that 20 of 476 cases required surgery. Of these, only four were malignant and all occurred within the first 8 years of observation.

**Conclusions:**

The study demonstrated that FNAC-rep is useless for the most of cases, hence it should only be considered for young adults having nodules with suspicious characteristics. Furthermore, a 10-year follow-up for ITNs is safe enough rather than a long-life follow-up.

**Supplementary Information:**

The online version contains supplementary material available at 10.1007/s40618-024-02510-3.

## Introduction

Thyroid nodule is a very common finding in the adult population worldwide, with a reported prevalence of up to 70%, especially in women and in the elderly [1]. This epidemiological evidence is mainly reconducted to the increasing use of ultrasound (US) and other imaging techniques in clinical practice over the last three decades, which identifies even non-palpable thyroid nodules. All these findings, although almost asymptomatic, may have a risk of malignancy and require an accurate evaluation [2]. The most accepted international guidelines recommend US assessment as the first step in thyroid nodule diagnostic work up [3–5]. However, US displays suboptimal accuracy for the diagnosis of malignant nodules, especially for some thyroid tumor subtypes such as medullary, follicular, and non-invasive follicular thyroid neoplasm with papillary-like nuclear features (NIFTP) [6–8]. Furthermore, the estimated risk of nodule malignancy is variable based on the risk stratification system used [9]. Beyond neck US, the mainstay diagnostic tool to refer a patient to surgery is the fine needle aspiration citology (FNAC) [3–5].

This methodology provides a cellular sample from the suspicious nodule, that is examined by the pathologist who assesses the nodule risk of malignancy based on its cyto-nuclear and cyto-architectural features [10].

Two systems for thyroid cytopathology classification are mainly used in clinical practice worldwide: Bethesda System for Reporting Thyroid Cytopathology (BSRTC) and Italian consensus for the classification and reporting of thyroid cytology (ICCRTC) [11–12]. These cytological classifications provide a clear diagnostic-therapeutic address to all their categories except for one.

Indeed, BSRTC includes “atypia of undetermined significance” (AUS) and ICCRTC “low-risk indeterminate lesion” (TIR3A) that are the only categories without a sharp malignancy meaning with the indication for FNAC repetition (FNAC-rep) [3–5; 11–12]. Despite these two systems recommend the use of these categories in less than 10% of diagnoses [12–13], in clinical practice their incidence is much higher reaching up to 30% [14–16]. Hence, nodules within these cytological classes, although with a relatively low risk of malignancy (10–15%), represent a heavy psychological burden for patients and their management and may have legal implications. Given the increasing number of patients with thyroid nodules diagnosed with TIR3A/AUS (hereinafter “indeterminate thyroid nodules” - ITNs), requiring a long-life active follow-up with one or more FNAC repetitions, these factors are emerging as a new crucial issue in daily clinical practice.

As a consequence, both American Thyroid Association (ATA) and European Thyroid Association (ETA) guidelines (in 2015 and 2023, respectively) recommend repeating FNAC in case of ITN diagnosis. Moreover, ETA recommend to “Repeat FNAC regardless of EU-TIRADS class”, while ATA do not provide any US criteria for repetition. In both cases the recommendations are supported by a very-low or low quality of evidence without clear indications about the timing to repeat the FNAC [3–5; 11–12]. In this scenario, FNAC-rep is empirically recommended within 6–12 months after the first FNAC (1st FNAC) and no data is available about the effect of delayed repetition on patient prognosis in terms of risk of nodule malignancy. On the other hand, no robust data are available in the literature regarding the long-term ITN follow-up.

Nowadays, the epidemiological relevance of the issue, its implications in clinical practice, and the absence of strong evidence on the actual usefulness and timing of repeated FNAC in patients with diagnosis of ITN require further urgent investigations.

Taking into account, the aforementioned issues, the aims of the study were to inquire the clinical usefulness of repeating FNAC in ITNs in terms of changing management indications and perform an evaluation of a long-term follow-up of a large cohort of ITNs.

## Methods

### Recruiting centres and clinical context

Two high-volume tertiary institutions for thyroid disease conducted the study: CTO Andrea Alesini Hospital in Rome (Italy) and Garibaldi Nesima Hospital in Catania (Italy). These centres perform more than 1000 FNACs per year applying the same standard diagnostic–therapeutic procedures for patients with thyroid nodules. All patients who refer to these institutions for thyroid evaluation undergo a general thyroid hormonal assessment (i.e., TSH, plus FT3 and FT4 when indicated, and Calcitonin) and US evaluation performed by endocrinologists with at least 10 years of experience. Thyroid nodules undergo FNAC according to main national and international guidelines [3–5]. Cytology smears are evaluated by an expert in thyroid field cytopathologist and classified according to ICCRTC. When the nodular lesion is diagnosed as TIR3A, FNAC is repeated at least six months later the first biopsy. In case of TIR3B, TIR4 or TIR5 (in the first or in the second FNAC), patients are referred to surgery. Whether FNAC-rep confirms diagnosis of TIR3A, clinical and US follow-up continues. In case of changes in clinical picture or US features, another FNAC is performed. Anyway, patients with double TIR3A at FNAC are referred to surgery according to guidelines, based on the evolution overtime of the clinical and US picture, and on the patient preferences. All patients sign informed consent and privacy forms for inclusion in clinical studies.

## Case selection and data collection

We extracted patients who underwent to FNAC from January 2015 to April 2024, from the two institutional databases. Inclusion criteria were: (1) 1st FNAC performed in one of the involved institutions; (2) 1st FNAC report of TIR3A; (3) The FNAC-rep performed in the same center of the first one; (4) Serum calcitonin in the normal range. Exclusion criteria were: (1) Diagnostic tests and follow-up performed in another clinical setting; (2) 1st FNAC report different than TIR3A; (3) TSH levels below 0.5 mcUI/ml; (4) Toxic thyroid nodules at scintigraphy.

We collected the following data for each patient included: Recruiting center; Sex; Age (years); BMI; US features at the time of 1st FNAC, such as, maximum diameter (millimeters), margins (i.e.; regular or irregular), echogenicity (i.e.; iso-anechoic, isoechoic, hypoechoic), and calcifications (i.e.; absent, micro-calcifications, macro-calcification); time for repetition (months); occurrence of margin, echogenicity, and calcification changes between 1st FNAC and FNAC-rep; variation in maximum diameter (millimeters); report of the FNAC-rep; Time since the first observation (months); Surgery (i.e.; yes or not); Pathology report (i.e.; benign or malignant); and Status of the follow-up at the last evaluation (i.e.; in progress, leave the follow-up, referred to surgery with subsequent benign pathology report, referred to surgery with subsequent malignant pathology report). We further categorized the variable “report of the FNAC-rep” in two other variables: “Different diagnosis” (i.e.; yes or not) and “Change in management indications” (CMIs) (i.e.; yes or not). We further categorized the variable “Status of the follow-up at the last evaluation” into another variable called “Event” (i.e.; 1 = “referred to surgery with subsequent malignant pathology report”; 0 = all the other conditions).

### Reference standard

The ICCRTC system is reliably comparable with the BSRTC. Both systems have a non-diagnostic category or with aspiration of cystic fluid only (i.e., TIR1/TIR1C– Class I), a benign/nonmalignant category (i.e., TIR2– Class II), a suspected for malignancy category (i.e., TIR4– Class V), and a malignant category (i.e., TIR 5 - Class VI). Furthermore, each system provides two indeterminate categories which according to the latest edition of BSRTC correspond as follows: TIR3B - Class IV plus part of Class III (subgroup with “nuclear atypia”), and TIR3A– part of Class III (subgroup with “other types of atypia”). The latter categories were the main focus of our study, therefore with the acronymous ITN we will refer to TIR3A and Class III (subgroup with “other types of atypia”) categories.

### Statistical analysis

Continuous variables were expressed as medians and interquartile range (IQR), categorical variables were expressed as numbers or percentages. Continuous variables were analyzed with a Test-T Student or Mann-Whitney U-Test, whereas categorical variables were compared by Chi-squared test or Fisher’s exact test, when appropriated. The binomial test was applied to evaluate whether in our cohort the number of cases with FNAC-rep report different from the 1st FNAC and the number of cases with changes in management indications following the FNAC-rep were statistically significant or not. We performed multiple univariate regression logistic analyses to investigate factors independently related to a FNAC-rep that changed indications in management of ITNs. Then, we searched the best fit multivariate logistic regression model using the putative predictors selected by univariate analyses. The overall time from the first observation of nodule to event was estimated using Kaplan-Meier curves. Cumulative hazard curve was also provided. A p-value of < 0.05 was considered to define statistical significance. All statistical analyses were performed with Jamovi software version 2.3.

## Results

**Fig. 1:**
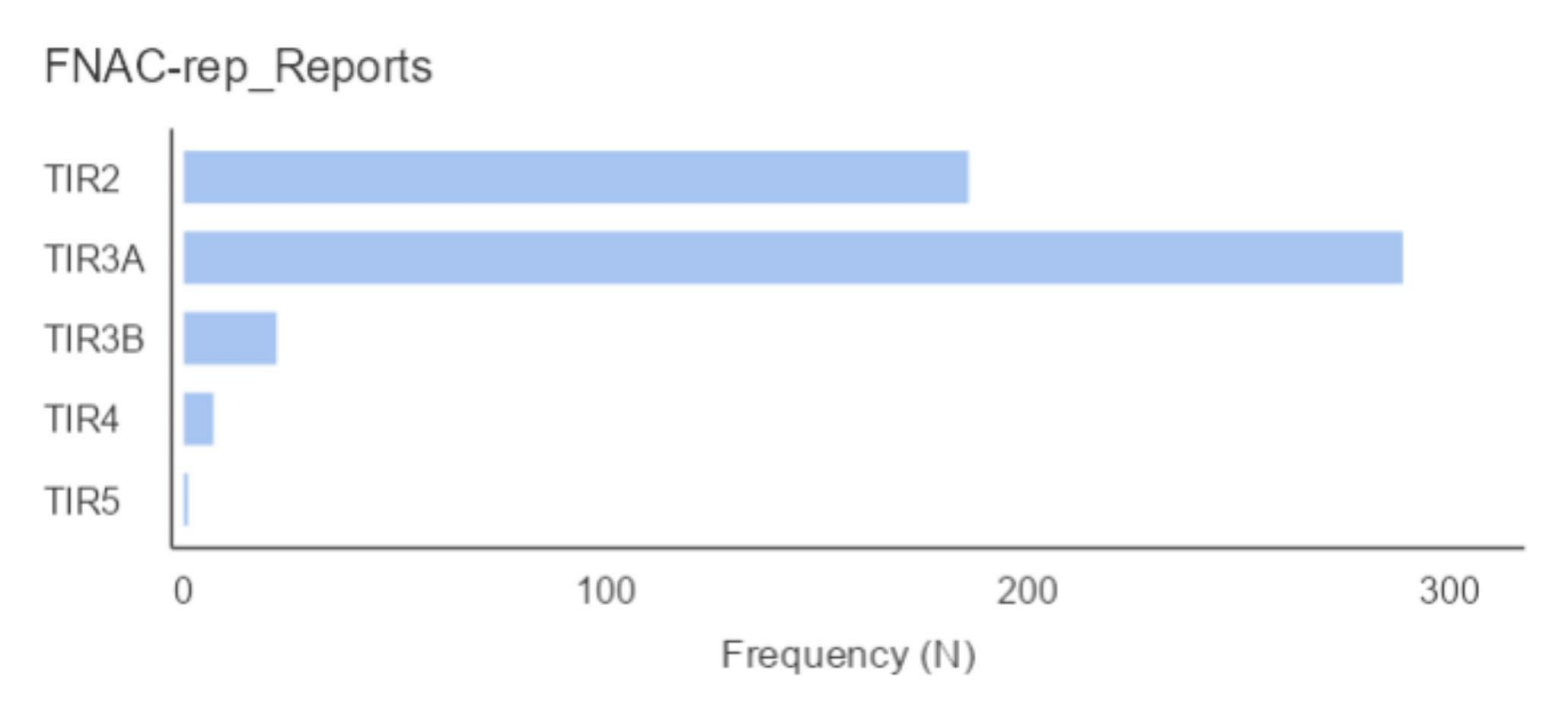
FNAC-rep reports

We included a total of 506 patients, 429 from Garibaldi Nesima Hospital (84.8%) and 77 from CTO Andrea Alesini Hospital (15.2%). There were 414 females (81.8%) and 92 males (18.2%) with a median age of 56 years (47–64) and a median BMI of 27.1 Kg/m^2^ (23.8–30.8). The median maximum diameter of nodules biopsied for the first time was 18 mm (14–24), their echogenicity was assessed as follows: 21.4% iso-anechoic, 44.2% isoechoic, and 34.4% hypoechoic. Calcifications were reported only in 7.4% of cases of which 6.2% were macro-calcifications, and 1.2% were micro-calcifications. The margins were irregular in the 3.1% of cases. The median time for FNAC repetition was 18 months (11–40) and the median variation in maximum diameter between the 1st FNAC and the FNAC-rep was + 1 mm. The latter ranged from − 11 mm to + 40 mm (Table 1). Cytology reports of FNAC-reps were 186 TIR2 (36.8%); 290 TIR3A (57.3%), 22 TIR3B (4.3%), 7 TIR4 (1.4%), and only one TIR5 (0.2%) (Fig. 1).

**Table 1:** Descriptive analysis of the sample included in the study

*N*. of cases	506
Recruiting center CTO Andrea Alesini Garibaldi Nesima	15.2% 84.8%
Age (years) (median ± IQR)	56 (47–64)
Sex Male Female	18.2% 81.8%
BMI (Kg/m^2^)	27.1 (23.8–30.8)
Maximum diameter (median ± IQR)	18 (14–24)
Echogenicity Iso-anechoic Isoechoic Hypoechoic	21.4% 44.2% 34.4%
Calcifications Absent Macro Micro	92.6% 6.2% 1.2%
Margins Regular Irregular	96.9% 3.1%
Time for repetition (months) (median ± IQR)	18 (11–40)
Variation in maximum diameter (mm)	+ 1 (− 11; +40)
Echogenicity change No Yes	79.9% 20.1%
Margins change No Yes	98.3% 1.7%
Calcifications occurrence No Yes	97.8% 2.2%
Cytology reports of FNAC-repetition TIR2 TIR3A TIR3B TIR4 TIR5	36.8% 57.3% 4.3% 1.4% 0.2%
Management after the FNAC-repetition Unchanged Changed	94.1% 5.9%

**Fig. 2:**
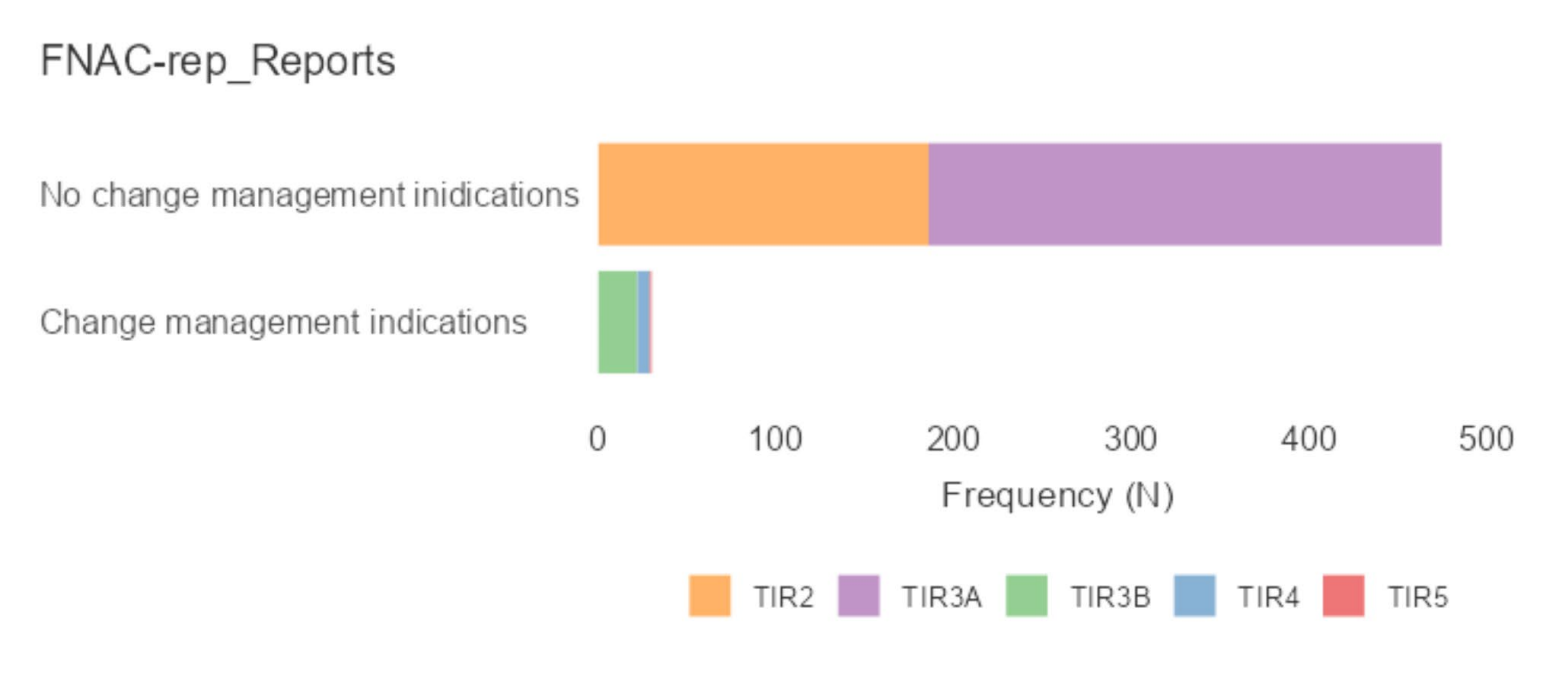
FNAC-rep reports divided based on any changes in management indications

Overall, 216 cases (42.7%) in the FNAC-reps showed reports different than the first one, assessed as TIR3A. Despite this, only 30 cases (5.9%) of the total sample received a diagnosis that changed management indications (i.e., TIR3B, TIR4, or TIR5) (Fig. 2).

There were no difference comparing the two recruiting centers in terms of male/female sample composition (*p* = 1.0) and age of the sample (*p* = 0.21), instead tested males were more aged than females (p 0.003). The binomial test showed that the proportion of repeated FNAC with a change in the report was significant (*p* < 0.001), instead, the proportion of cases which changed their management indications was not significant (p 0.36).

We explored difference in terms of gender and age in variable CMIs. Chi-squared test did not show significant differences (p 0.09) in gender composition, instead Test-T showed significant lower age in patients with positive CMIs following FNAC-rep.

To identify clinical and/or ultrasound characteristics that independently determine CMIs, we performed logistic regression analysis in the 506 included patients (Table 2). Univariate logistic regression analyses revealed that the change in the suggested management after FNAC repetition was related to age (OR = 0.97; 95%CI = 0.94–0.99; *p* = 0.025), maximum diameter (OR = 0.88; 95%CI = 0.82–0.94; *p* < 0.001), margins (OR = 12.6; 95%CI = 4.14–38.24; *p* < 0.001), echogenicity (hypoechoic vs. iso-anechoic: OR = 8.07; CI95%= 1.9–35; *p* = 0.005| hypoechoic vs. isoechoic: OR = 6.65; 95%CI = 2.5–17.9; *p* < 0.001) and margin changes (OR = 6.68; 95%CI = 1.26–35.34; p 0.025). There was no statistical significance for sex, calcifications (micro or macro), time for repetition, delta of maximum diameter, echogenicity changes, and calcification changes.

**Table 2:** Univariate and Multivariate analysis according to the change of the management indications after the FNAC-repetition

	Univariate analyses			Multivariate analysis		
	Odds ratio	95% CI	p value	Odds ratio	95% CI	p value
Age	0.97	0.95–0.99	**0.025**	0.97	0.95–0.99	**0.04**
Sex	0.30	0.07–1.30	0.110			
Maximum diameter	0.88	0.82–0.94	**< 0.001**			
Echogenicity Hypoechoic vs. iso-anechoic Hypoechoic vs. isoechoic	8.07 6.65	1.90–35 2.50–17.90	**0.005** **< 0.001**	5.9 5.2	1.32–26.2 1.87–14.5	**0.02** **0.002**
Margins	12.60	4.14–38.24	**< 0.001**	5.6	1.7–18.1	**0.004**
Margins change	6.68	1.26–35.34	**0.025**			
Calcifications Absent vs. Macro Absent vs. Micro	2.57 < 0.001	0.83–7.90 0.0 - >100	0.101 0.989			
Time for FNAC repetition	0.98	0.97–1.0	0.174			
Variation in maximum diameter	0.99	0.98–1.03	0.171			
Echogenicity change	0.64	0. 19–2.25	0.496			
Calcifications occurence	4.17	0.67–26.0	0.127			

**Fig. 3:**
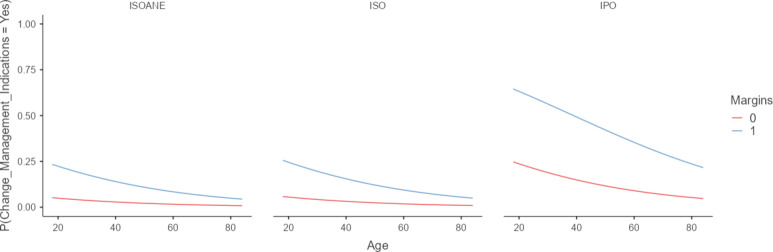
Risk of change management indications following FNAC repetition according to age, margins, echogenicity

Furthermore, considering the results of the univariate analyses, a multivariate logistic regression (Table 2) was performed. The best fit model (R^2^ = 0.16; AIC = 200; *p* < 0.001) included age (OR = 0.97; 95%CI = 0.95–0.99; *p* = 0.04), margins (OR = 5.6; 95%CI = 1.7–18.1; *p* = 0.004), and echogenicity (hypoechoic vs. isoechoic: OR = 5.2; 95%CI = 1.87–14.5; *p* = 0.002| hypoechoic vs. iso-anechoic: OR = 5.9; 95%CI = 1.32–26.2; *p* = 0.02) (Fig. 3).

**Fig. 4:**
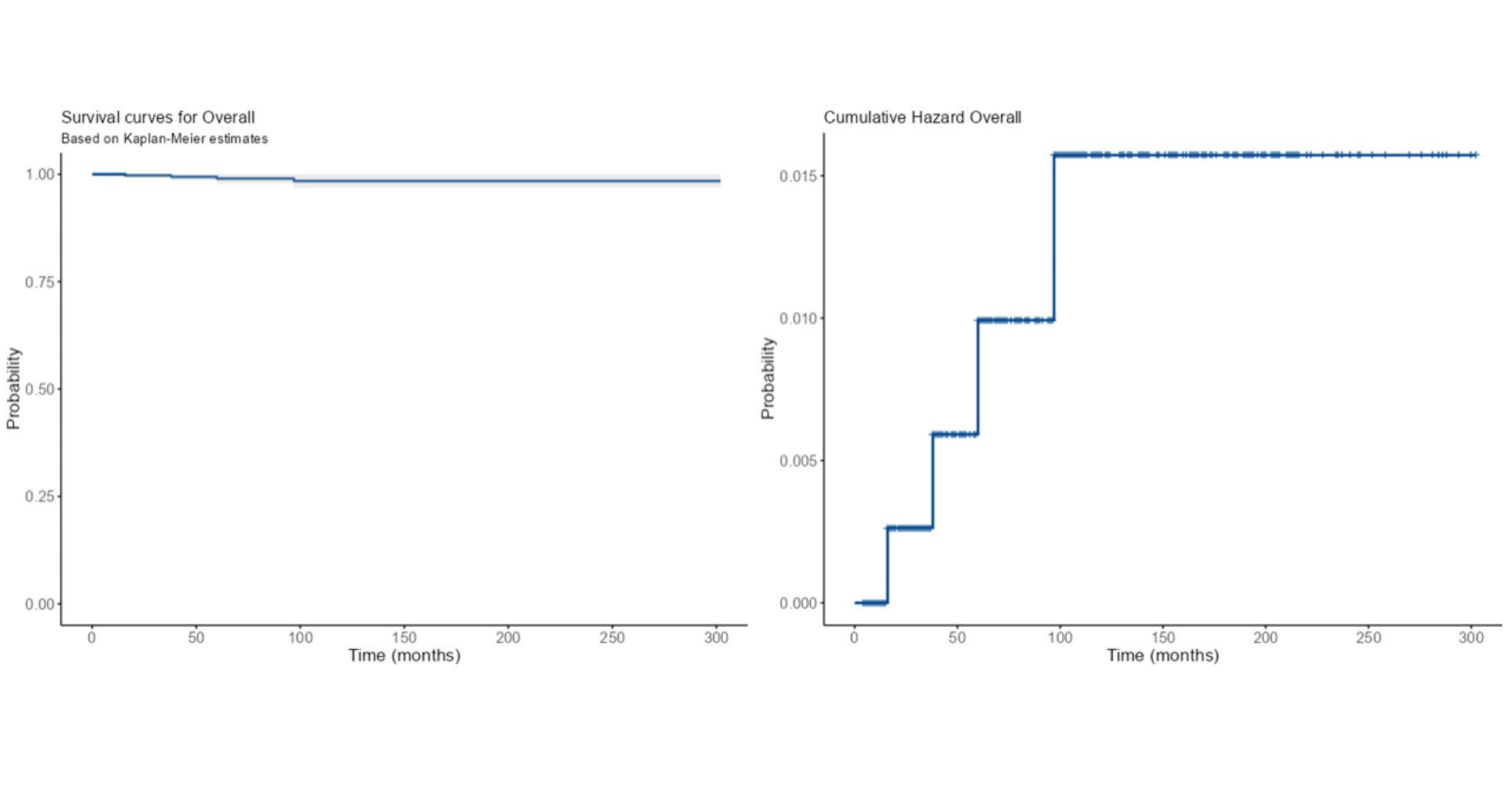
Kaplan-Meier and Cumulative Hazard curves

We further analyzed follow-up data from patients with a double FNAC that did not lead to a CMIs. The median follow-up period of nodules was 80 months, ranging between 4 and 300 months. Twenty patients were thyroidectomized of which 16 resulted with benign pathology and 4 with malignant pathology. Specifically, 9 patients with cytological diagnosis of TIR2 on FNAC-rep, one of whom had malignant pathology, and 11 patients with cytological diagnosis of TIR3A on FNAC-rep, 3 of whom had malignant pathology. The malignant cases ranged from 19 to 38 years and all the diagnosis occurred within 97 months (≈ 8 years), with a median follow-up of 49 months. The estimated cumulative hazard overall was of 0.015 at 100 months. The mean overall time from the first observation of nodule to event estimated by Kaplan-Meier curve was 289 months (Fig. 4).

Finally, the other 456 cases showed no clinical or ultrasound changes requiring surgery during their long-term follow-up.

## Discussion

The management of ITN is a relevant issue in daily clinical practice; however, there is little evidence on: the usefulness of repeating FNAC, its timing, and the ultrasound features that could help further select who should repeat it. The latter are critical questions especially in the current era of evidence-based medicine and cost-effectiveness of diagnostic and follow-up processes. In this study we investigated the actual usefulness of repeating FNAC in ITNs in terms of changing in management indications and performed an evaluation of a long-term follow-up of a large cohort of ITNs. The study findings deserve several points of discussion with important implications for clinical practice.

The included population was uniform in both institutions; hence we can affirm that the inclusion criteria were rigorous. Moreover, the large part of the sample included females and the median BMI indicates diffuse overweight in the selected patients. These data support existing literature on epidemiology of nodular thyroid goiter and confirm the reliability of our sample [17–19].

The wide distribution of nodule diameter and US features indicated that nodules were selected for FNAC both by a size criterion (larger nodules) and US features, in accordance with international guidelines.

One of the most interesting findings of the study was the high percentage of FNAC-reps with a different diagnosis than the 1st FNAC diagnosed as ITN. Of these, almost all FNAC-reps were assessed as benign. The discrepancy between the two examinations may be justified by the aspiration of different cellular populations during the procedures or by the similar cytological patterns between ITNs and benign lesions. In fact, discrimination of cytological alterations between benign and undetermined risk lesions is not always clearly defined [20]. Despite the relevance of this figure, it did not translate into an effective change in the patient’s clinical management, given the persistence of the previous cytology examination which reported an undetermined risk of malignancy. Therefore, in the most of cases, repeating FNAC was not helpful to improve the clinical management of ITNs.

When we further investigated factors independently related to CMIs following the FNAC-rep, we found that age, margins, and echogenicity were significantly related to it. Age was inversely related to an increased risk of malignancy at FNAC-rep (hence, CMIs) (Fig. 4). In accordance with previous evidence, thyroid nodules in the young adult population are more likely to be malignant than in the elderly [21–23].

Irregular margins and hypo-echogenicity were the other factors related to CMIs of ITNs. Both factors are fully recognized as associated with thyroid carcinoma. Irregular margins suggest malignant infiltration of adjacent thyroid parenchyma with no pseudocapsule formation, instead hypo-echogenicity suggest hypercellularity and poor colloid in the nodule tissue [24]. Therefore, although overall FNAC-rep seems to be useless, we may identify subgroups of patients to consider for FNAC-rep in order to personalize ITN management.

Hence, we propose to consider FNAC-rep only for young adult patients, up to a maximum of 55 years and/or having nodules with suspicious US characteristics. In this category, FNAC-rep should be performed after at least 3–6 months to allow inflammation and tissue repair recovery occurring after the first FNAC, since the risk of malignancy of the FNAC-rep does not change over time, as demonstrated by regression analyses. It is interesting to note that calcifications are not significantly related to CMIs at FNAC-rep, because microcalcifications are present in smears at higher risk of malignancy, which are rarely diagnosed as ITN and are present in our series at very low rate.

Finally, discussing the long-term follow-up of patients with FNAC-rep diagnosis of ITN or benign, the estimated cumulative risk of event (i.e., diagnosis of thyroid carcinoma after surgery) was very low and the mean overall time from the first observation of the nodule to event was very long. These data support the need for a shorter and less strict follow-up of patients with ITNs. Hence, we suggest reducing frequency of follow-up after ten years of observation and, in turn, it would be reasonable to stop follow-up in patients aged over 70 years. Moreover, it is necessary to keep in mind other diagnostic procedures, such as ^99m^Tc-MIBI scintigraphy, which have also been shown to be useful in improving the management of ITNs [25].

A final consideration raises from the several discussed evidence about the terminology currently used: TIR3A and AUS nodules (the latter exclusively for the subgroup with “other types of atypia”) should be more realistically defined as “probably benign lesions” rather than lesions of undetermined risk/significance.

This study opens to new approaches for management of patients with ITNs, despite this, it is necessary to report its limitations: (1) the retrospective observational design and (2) the lack of histological examination for the most of patients. However, the findings are strengthened by real-world data from high-volume tertiary thyroid disease centres, large sample sizes, and long-term follow-up.

## Conclusions

ITN management is a relevant issue of clinical practice. Our study demonstrated that FNAC repetition is useless for the most of cases, hence it should only be considered for young adults having nodules with US suspicious characteristics. Furthermore, a ten-years follow-up for ITNs is safe enough rather than a long-life follow-up. Future international guidelines should take these findings into account.

## Conflict declaration

## Electronic supplementary material

Below is the link to the electronic supplementary material.

Supplementary Material 1

## Data Availability

The data sets used and/or analyzed during the current study are available from the corresponding author on reasonable request.
