# Supplementary material for: Clinical management of indeterminate thyroid nodules needs to be revisited. New evidence for a personalized approach to the problem
Source: J Endocrinol Invest. 2024 Dec 4;48(4):885–93. doi: 10.1007/s40618-024-02510-3 (PMC11950127; doi:10.1007/s40618-024-02510-3)
Supplement: Supplementary file 1 — Supplementary Material 1 [file 40618_2024_2510_MOESM1_ESM.docx]

**Figure 1.** FNAC-rep reports.

**Figure 2.** FNAC-rep reports divided based on any changes in management indications.

**Figure 3.** Risk of change management indications following FNAC repetition according to age, margins, echogenicity.

**Figure 4.** Kaplan-Meier and Cumulative Hazard curves
